# A replication of the relationship between adversity earlier in life and elderly suicide rates using five years cross-national data

**DOI:** 10.5249/jivr.v4i1.65

**Published:** 2012-01

**Authors:** Ajit Shah

**Affiliations:** ^a^University of Central Lancashire, Preston, United Kingdom and Consultant Psychiatrist, West London Mental Health NHS Trust, London, United Kingdom.

## Abstract

**Background:**

Although life-long adversity has been suggested as a protective factor for elderly suicides, studies examining protective factors for elderly suicides are scarce. A cross-national study examining the relationship between elderly suicide rates and several proxy measures of adversity earlier in life was undertaken to replicate earlier findings by using five consecutive years data on elderly suicide rates from a more recent data set and by adding two more proxy measures of adversity early in life.

**Methods:**

The relationship between elderly suicide rates and five proxy measures of adversity earlier in life was examined using data from the World Health Organization and the United Nations data banks with Spearman’s correlation coefficient. The five proxy measures of adversity early in life were: the percentage of children under the age of 5 years who were under weight, the percentage of children under the age of 5 years who were under height, the percentage of infants with low birth weight babies, the percentage of the general population with sustainable access to improved sanitation and the percentage of the general population with sustainable access to an improved water source.

**Results:**

Generally, elderly suicide rates were lower in countries with higher adversity early in life. The only exceptions were in females aged 75+ years where this association only approached statistical significance for the percentage of children under the age of 5 years who were under weight and the percentage of children under the age of 5 years who were under height.

**Conclusions:**

The current study using a more recent data set and a five years data on elderly suicide rates along with two additional proxy measures of adversity early in life was able to replicate the findings of the earlier study. This suggests that the findings of the earlier study were accurate and robust.

## Introduction

The elderly population size is increasing in most countries.^1^Traditionally, over the last two to three decades, suicide rates increased with ageing.^2^A recent cross-national study of 62 developing and developed countries reported an increase in suicide rates with ageing in males and females in 25 and 27 countries respectively,^3^although in the remaining countries suicide rates were either higher or similar in the younger age groups. Thus, suicides in the elderly are an important public health concern. Comprehensive understanding of the substantial worldwide variation in population patterns of suicide may be critical for developing prevention programmes.^4^While much is known about individual level risk factors,^5-7^less is known about protective factors.^3,8^Moreover, detailed knowledge of protective factors may also have greater public health relevance for universal prevention strategies.^3,8^

Elderly suicide rates have been shown to be low in countries with low socio-economic status including those with greater income inequality.^9,10^Life expectancy is also reduced in these countries.^9,10^Therefore, given that suicide rates generally increase with age, it is possible that in some countries with low elderly suicide rates fewer people will reach the age of increased risk of suicide due to reduced life expectancy.^10^Moreover, selective survival of those at reduced risk for suicide due to genetic or constitutional factors may further compound this trend.^10^Furthermore, those who do may be at reduced risk of suicide in old age because they may be able to tolerate extra hardship in old age better due to exposure to life-long adversity.^11,12^For example, elderly African Americans and native Americans (Indians) have low suicide rates ^13^ and this has been attributed to a life-long history of socio-economic deprivation.^13^In a recent cross-national study, elderly suicide rates were inversely associated with proxy markers of adversity early in life including the percentage of children under the age of 5 years who were under weight, the percentage of children under the age of 5 years who were under height and percentage of infants who were of low birth weight.^14^Another cross-national study did not find a relationship between elderly suicide rates and two measures of current economic adversity: average annual growth rate and average annual change in the consumer price index over a long-term period.^15^These studies had used single year data on suicide rates and, therefore, the findings may have been spurious as suicide rates can randomly fluctuate year on year.^16^Therefore, a cross-national study examining the relationship between elderly suicide rates and several proxy measures of adversity earlier in life was undertaken using five consecutive years data and more recent data on elderly suicide rates to attempt to replicate earlier findings.

## Methods

Data on elderly suicide rates for males and females in the age-bands 65-74 years and 75+ years for the latest five years were ascertained from the World Health Organization (WHO) (www.who.int/ whosis/database/mort /table1.cfm). The median (range) of the latest available year for data on suicide rates was 2005 (1983-2007); this data set was more recent than that used in the previous study.^14^The one-year average suicide rate was calculated by dividing the sum of the suicide rates for each of the five years by 5. Fuller details on the methodology of ascertaining elderly suicide rates is provided elsewhere.^17^

The United Nations Development Programme (UNDP) website(http://hdr.undp.org/en/media/HDI_2008_EN_Tables.pdf) provided data on the percentage of children under the age of 5 years who were under weight, the percentage of children under the age of 5 years who were under height, the percentage of infants with low birth weight babies, the percentage of the general population with sustainable access to improved sanitation and the percentage of the general population with sustainable access to an improved water source. These five variables were considered to be proxy measures of adversity earlier in life. They have been considered to be proxy measures of adversity earlier in life in our previous study^14^and was considered to have face validity.^14^Although these measures may have changed over time, they are likely to have improved and may still give an indication of adversity earlier in life. The three variables of the percentage of children under the age of 5 years who were under weight, the percentage of children under the age of 5 years who were under height and the percentage of infants with low birth weight babies have previously been used as proxy measures of adversity early in life; they were available for an unspecified year between 1996 and 2004. The two variables of the percentage of the general population with sustainable access to improved sanitation and the percentage of the general population with sustainable access to an improved source of water were considered additional proxy measures of adversity early in life; they were available for the year 2004.

The relationship between elderly suicide rates and the five proxy measures of adversity earlier in life was examined with Spearman’s rank correlation (rho).

## Results

Table 1 illustrates the relationship between suicide rates and the proxy measures of adversity earlier in life. Suicide rates in males and females in both the elderly age-bands were significantly correlated with percentage of infants with low birth weight (negative), the percentage of the general population with sustainable access to improved sanitation (positive) and the percentage of the general population with sustainable access to an improved source of water (positive). Suicide rates in males in both the age-bands were significantly correlated with the percentage of children under the age of 5 years who were under weight (negative) and the percentage of children under the age of 5 years who were under height (negative); in females this relationship was significant in the 65-74 year age-band, and only approached significance in females aged 75+ years.

**Table:** Table 1:**The relationship between suicide rates and proxy measures of adversity earlier in life**

	% Children Under Weight N=46	% Children Under Height N=48	% Infants With Low Birth N=82	% of Total Population with improved Sanita-tion N=60	% of Total Population with improved water source N=69
Suicide Rates:					
Males 65-74 years	Rho=-0.48	Rho=-0.46	Rho=-0.48	Rho=+0.4	Rho=+0.3
	P=0.001	P<0.0001	P<0.0001	P=0.002	P=0.012
Males 75+ years	Rho=-0.46	Rho=-0.5	Rho=-0.37	Rho=+0.45	Rho=+0.35
	P=0.001	P<0.0001	P=0.001	P<0.0001	P=0.003
Females 65-74 years	Rho=-0.37	Rho=-0.34	Rho=-0.44	Rho=+0.52	Rho=+0.4
	P=0.012	P=0.002	P<0.0001	P<0.0001	P=0.001
Females 75+ years	Rho=-0.28	Rho=-0.25	Rho=-0.39	Rho=+0.36	Rho=+0.28
	P=0.064	P=0.094	P<0.0001	P=0.02	P=0.02

## Discussion

Some methodological issues need consideration. First, cross-national data on suicide rates should be viewed cautiously because: data are not available from all countries;^18,19^the validity of this data is unclear;^19,20^the legal criteria for the proof of suicide vary between countries and between different regions within a country;^19^some countries, particularly low-income countries, may have poor death registration facilities;^2^and, cultural and religious factors and stigma attached to suicide may lead to under-reporting of suicides.^19,21^Second, several variables were assumed to be proxy measures of adversity earlier in life. Third, data on suicide rates were for a variable time period and may have introduced biased in the analysis. Fourth, caution should be exercised in assuming the direction of causal relationships in this cross-sectional and ecological study because of ecological fallacy. Nevertheless, the best and the latest available data were ascertained from the WHO and UNDP data banks.

The observed correlations between elderly suicide rates and the five proxy measures of adversity earlier in life may be explained by the above methodological difficulties. However, it is possible that the correlations are genuine because they confirm earlier reports of an association between life-long adversity and lower elderly suicide rates,^11-14^The current study was different from the earlier study^14^because: it used an annual average of five years data on elderly suicide rates; it used a more recent data set on suicide rates; and it used two other proxy measures of adversity early in life. This suggests that the findings of the earlier study were robust and accurate.
